# Metabolomic Profiling of the Responses of Planktonic and Biofilm *Vibrio cholerae* to Silver Nanoparticles

**DOI:** 10.3390/antibiotics11111534

**Published:** 2022-11-02

**Authors:** Anaid Meza-Villezcas, Rommel A. Carballo-Castañeda, Aldo Moreno-Ulloa, Alejandro Huerta-Saquero

**Affiliations:** 1 Departamento de Bionanotecnología, Centro de Nanociencias y Nanotecnología, Universidad Nacional Autónoma de México, Ensenada 22860, BC, Mexico; 2 Departamento de Innovación Biomédica, Centro de Investigación Científica y de Educación Superior de Ensenada, Ensenada 22860, BC, Mexico; 3 Departamento de Microbiología, Centro de Investigación Científica y de Educación Superior de Ensenada, Ensenada 22860, BC, Mexico

**Keywords:** cholera, planktonic cells, biofilm, silver nanoparticles, AgNPs, tandem mass spectrometry, MS2, metabolic perturbation

## Abstract

*Vibrio cholerae* causes cholera and can switch between planktonic and biofilm lifeforms, where biofilm formation enhances transmission, virulence, and antibiotic resistance. Due to antibiotic microbial resistance, new antimicrobials including silver nanoparticles (AgNPs) are being studied. Nevertheless, little is known about the metabolic changes exerted by AgNPs on both microbial lifeforms. Our objective was to evaluate the changes in the metabolomic profile of *V. cholerae* planktonic and biofilm cells in response to sublethal concentrations of AgNPs using MS2 untargeted metabolomics and chemoinformatics. A total of 690 metabolites were quantified among all groups. More metabolites were significantly modulated in planktonic cells (*n* = 71) compared to biofilm (*n* = 37) by the treatment. The chemical class profiles were distinct for both planktonic and biofilm, suggesting a phenotype-dependent metabolic response to the nanoparticles. Chemical enrichment analysis showed altered abundances of oxidized fatty acids (FA), saturated FA, phosphatidic acids, and saturated stearic acid in planktonic cells treated with AgNPs, which hints at a turnover of the membrane. In contrast, no chemical classes were enriched in the biofilm. In conclusion, this study suggests that the response of *V. cholerae* to silver nanoparticles is phenotype-dependent and that planktonic cells experience a lipid remodeling process, possibly related to an adaptive mechanism involving the cell membrane.

## 1. Introduction

*Vibrio cholerae* causes cholera, an acute diarrheal infection initiated by ingesting contaminated drinking water and food. Cholera is a significant public health concern, affecting 1–4 million people annually, with 21,000 to 143,000 fatal cases [1,2,3]. *V. cholerae* is a natural inhabitant of aquatic environments and lives as free-living cells (planktonic cells) or forming biofilms [4]. Biofilms are surface-attached microbial communities composed of microorganisms and an extracellular matrix that protect from harsh environmental conditions, including predation and physicochemical changes. Biofilms are associated with a hyper-infectious phenotype [5] and antibiotic resistance [6,7,8,9] and it is estimated that approximately 80% of chronic and recurrent infections in the human body are due to biofilms [2,4]. 

Among pathogenic bacteria such as *V. cholerae* the rate of antimicrobial resistance exceeds the development of new antibiotics [10,11,12,13]; therefore, finding new substances with antimicrobial properties such as metal nanoparticles (NPs) could be an effective alternative. The antimicrobial activity of silver nanoparticles (AgNPs) has been amply do-cumented and AgNPs are among the most widely commercialized nanomaterials in health care [14,15,16,17,18,19,20]. In a previous report, nanocomposite zeolite-embedded silver nanoparticles (ZEO-AgNPs) were evaluated in *V. cholerae* planktonic cells and two different types of biofilms; the pellicle biofilm (PB) formed between the air–liquid interface, and the surface-attached biofilm (SB) developed at the solid–liquid interfaces [21]. The ZEO-AgNP nanocomposite inhibited PB formation, prevented SB, and eliminated planktonic cells. Specifically, the relative abundance of the outer membrane proteins OmpA and OmpW also differed among the PB and SB, hinting at microbial phenotype-dependent responses to nanoparticle treatment [21]. Yet, studies evaluating the metabolic response to AgNPs by different microbial phenotypes (i.e., planktonic vs. biofilm lifeforms) are lacking. 

Metabolomics-based approaches allow studying the microbial metabolism responses to external stimuli, thereby providing insights into the specific cell responses under different conditions [22,23,24]. In biofilm overproducing variants of *P. fluorescens*, nuclear magnetic resonance (NMR)-based metabolomics revealed changes in the production of amino acids and certain metabolites through the glutathione pathway, which are hypothesized to play a role in redox and metal homeostasis [25]. Depending on the colony morphology of the variant, the biofilm responds differently to withstand environmental stresses. A combined GC-MS and H NMR metabolomic study on *P. fluorescens* under copper ion stress showed significantly different metabolic profiles among planktonic cells and biofilm phenotypes [26]. For instance, planktonic cells experienced oxidative stress and changes in the TCA cycle, glycolysis, pyruvate, nicotinate, and nicotinamide metabolism. At the same time, the biofilm response was dominated by shifts in exopolysaccharide-related metabolism, hinting at phenotype-dependent responses to external metal treatments.

In this work, we profiled the metabolomic responses of planktonic and biofilm *V. cholerae* lifeforms to AgNPs using mass spectrometry-based untargeted metabolomics and comprehensive chemoinformatics to gain insight into the phenotype-dependent response of the bacteria to AgNPs. 

## 2. Results

### 2.1. AgNP Characterization

**Figure 1:**
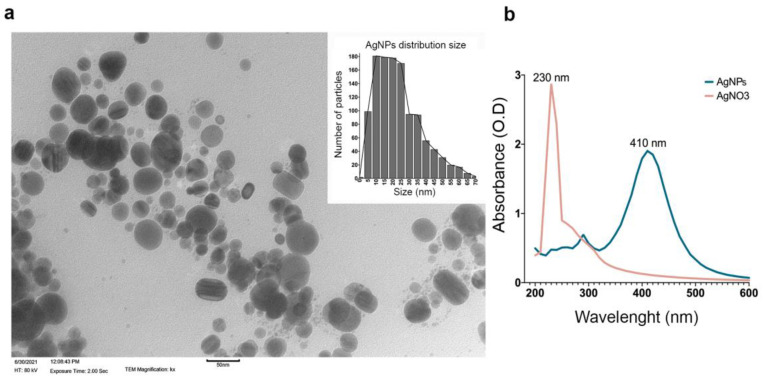
**Silver nanoparticles (AgNPs) characterization.** (**a**) Transmission electron microscopy of AgNPs. One bar represents 50 nm. AgNPs’ size distribution is shown in the insert. (**b**) UV–Vis profile of AgNPs and AgNO_3_.

AgNPs were synthesized following the protocol described by Vazquez-Muñoz [27] with some modifications. Representative images are shown in Figure 1a. TEM analysis revealed that the sizes of the nanoparticles were between 10 nm and 35 nm and were a spheroid shape. The AgNPs’ UV–Vis profile showed a single peak with a maximum absorbance at ʎ = 410 nm, typical for small spheroid AgNPs [28] (Figure 1b). An AgNO_3_ absorbance at ʎ = 230 nm was indicated (red spectra), and the AgNPs’ synthesis after dialysis and lyophilization lost the AgNO_3_, which was not reduced to AgNPs (blue spectra). The various syntheses showed a maximum of around 410 nm without AgNO_3_. Similarly, the absorbance intensity slightly varied in each synthesis, possibly associated with the AgNPs’ relative concentration (Figure 1b). The hydrodynamic size (HS) of the AgNPs ranged from 12 to 20 nm. The AgNPs’ Zeta potential was—6.2 mV (Figure S1). The synthesis weight percentage was calculated using concentrations of 1.8 ppm and 2.6 mg by a global chemical analysis performed by inductively coupled plasma-atomic emission spectroscopy ICP-OES, resulting in 89.3% of silver (Figure S2).

### 2.2. The AgNPs Showed a Different Antimicrobial Effect Depending on the Lifeform of Vibrio cholerae

**Figure 2:**
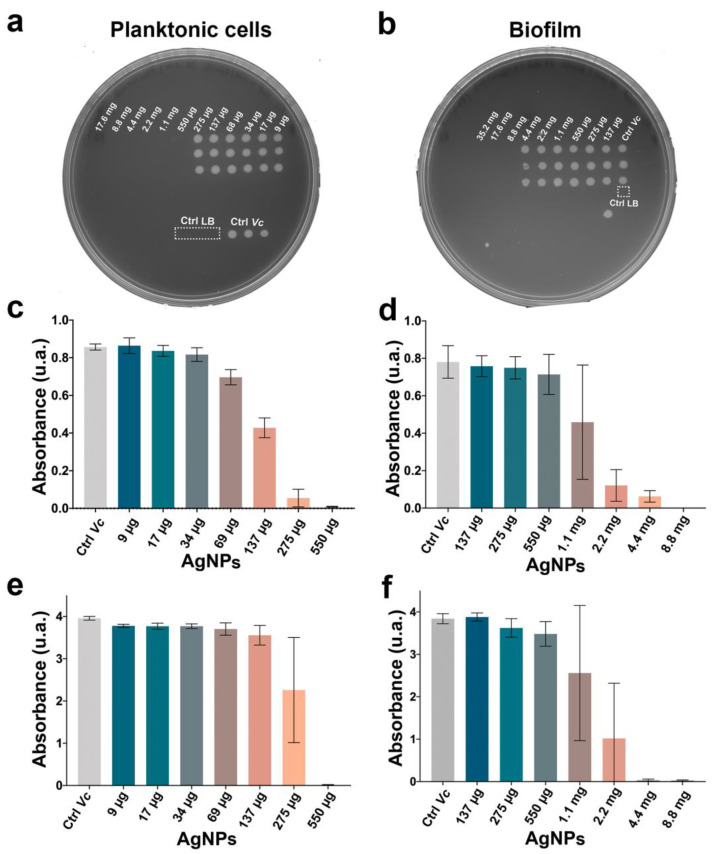
**AgNPs’ minimal bactericidal concentrations of planktonic cells (PL) and biofilms (BF).** By stamping (**a**,**b**), MTT assay (**c**,**d**), Turbidity (**e**,**f**). Three biological replicates (each one with three technical replicates). *V. cholerae* without treatment (*Ctrl Vc*); Lysogeny Broth (LB) media as a control with no bacterial contamination; MTT (3- (4,5-dimethylthiazol-2-yl) -2,5-diphenyltetrazolium bromide).

The antimicrobial properties of AgNPs were evaluated against *V. cholerae* wild-type (smooth variant) as planktonic cells as well as mature biofilms. The minimal bactericidal concentration (MBC) of the planktonic cells and the minimal biofilms’ bactericidal concentration (MBBC) was determined by the direct stamp of the treatments (Figure 2a,b). The bactericidal concentrations for the planktonic cells and biofilms were 550 µg/mL and 4.4 mg/mL of AgNPs, respectively. The minimal inhibitory concentration was determined by optical density (Figure 2c,d) and MTT assay (Figure 2e,f). For the planktonic cells, the MTT assay and the optical density were in agreement at 275 µg/mL, the concentration at which turbidity was no longer observed. On the other hand, the biofilms presented a broader range of inhibition, ranging from 1.1 mg to 4.4 mg, the latter concentration consisting of the three biological replicates. According to the results of the MTT assay (reducing the tetrazolium dye MTT 3- (4,5-dimethylthiazol-2-yl) -2,5-diphenyltetrazolium bromide to its insoluble formazan) and optical density (Figure 2c–f), the AgNPs’ minimal inhibitory concentration (MIC) for the biofilms was determined to be 4.4 mg/mL (Figure S3). 

Once the MBC and MBBC of *V. cholerae* in both lifestyles was established, the LD_50_ (lethal dose 50%) as a sublethal concentration was chosen for the subsequent experiments. Bacterial cultures of the planktonic cells and biofilms were exposed to concentrations of 275 µg/mL and 2.2 mg/mL, respectively.

### 2.3. AgNPs Differentially Modify the Metabolome of Vibrio cholerae According to its Phenotype

**Figure 3:**
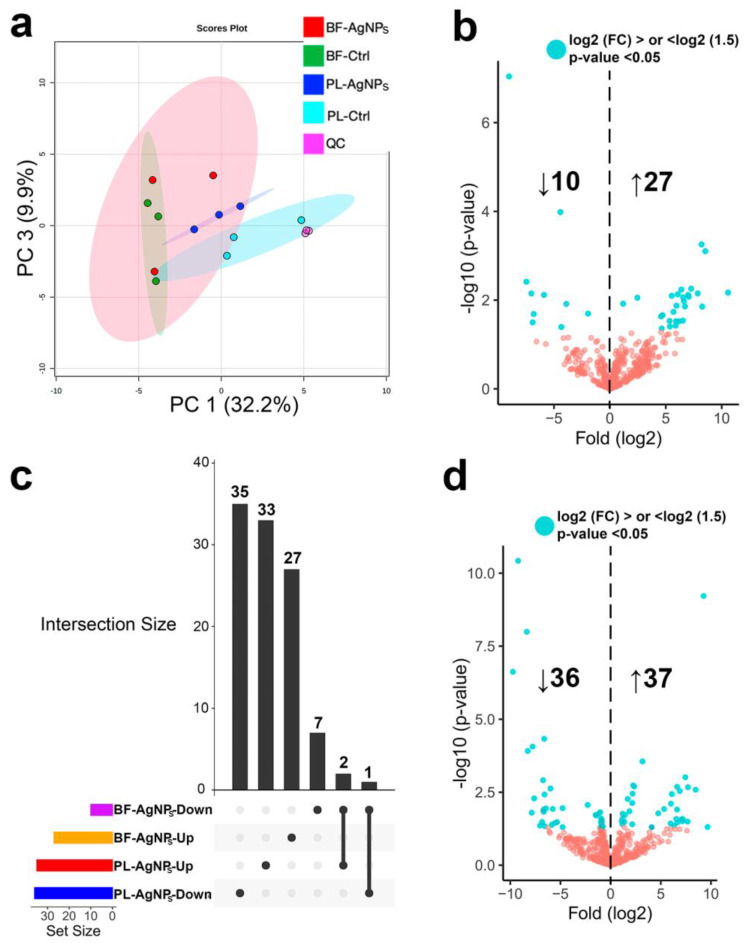
**Mass spectrometry-based untargeted metabolomics and chemoinformatic analyses of planktonic cell (PL) and biofilm (BF) *V. cholerae* exposed to silver nanoparticles (AgNPs).** (**a**) Principal Component Analysis (PCA) using the aligned and filtered features among the groups. Data as log-transformed without scaling. Shaded areas denote 95% confidence intervals. Volcano plot of the quantified features in (**b**) biofilm (BF-AgNPs/BF-Ctrl) and (**d**) planktonic (PL-AgNPs/PL-Ctrl) *V. cholerae* exposed to AgNPs. Blue dots indicate features significantly modulated by AgNPs treatment based on fold-change and *p*-value (Limma test) criteria. (**c**) Overlapping pattern (Upset plotting) of dysregulated metabolites by AgNPs under conditions where the interconnected points between the groups indicate that 3 of the 105 perturbed metabolite features were shared.

To globally visualize the metabolic responses of *V. cholerae* planktonic (PL) and biofilm (BF) phenotypes exposed to sublethal concentrations of AgNPs, we performed a principal component analysis (PCA), whereby we noted grouping the patterns among the datasets (Figure 3a). The BF and PL control groups were clustered away from each other, indicating differences in the basal metabolome between both microbial phenotypes. AgNPs perturbed metabolomes differently according to the phenotype and PL-treated cells clustered away from non-treated cells; however, the same was not true for treated and non-treated BF. The tight clustering of the quality control (QC) samples reflects the method’s stability and ensures that our observed changes have a biological and not technical origin.

To compare the feature abundance of the AgNPs-treated planktonic and biofilm groups against their respective controls (non-treated cells) and gain an insight into the specific metabolites altered by the treatment according to each microbial phenotype, the data were mined by various complementary chemoinformatic tools. Overall, we quantified with high confidence 619 metabolites across all groups, and the specific quantitative comparisons are illustrated as volcano plots (Figure 3b,d). More significant dysregulated metabolites (based on fold-change and *p*-value criteria) were found in the planktonic cells (35 up, 36 down) than in the biofilms (27 up, 10 down). 

To determine if AgNPs altered the production of the same metabolites between microbial lifeforms, we employed an overlapping pattern (UpSet plotting) as a data visualization method, containing all the dysregulated metabolites (Figure 3c). Only 3 of the 105 perturbed features were shared, suggesting a phenotype-dependent metabolic response of *V. cholerae* exposed to AgNPs. 

**Figure 4:**
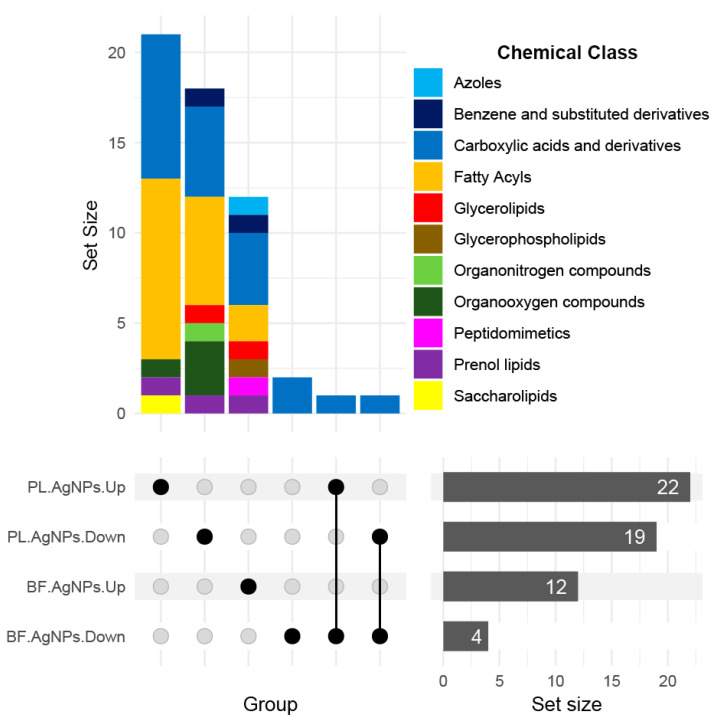
**Metabolites annotated at the chemical class level and altered by AgNPs under specific conditions.** Conditions (low, horizontal categories) are established based on lifeform (planktonic [PL] or biofilm [BF]) and change in direction (up or down modulated). Dark grey horizontal bars show the number of metabolites in each set. Dots indicate whether the metabolites are changed exclusively under one (simple dot) or more conditions (linked dots). Vertical bars represent the number of metabolites in each single or combined set and colors represent the chemical class’s re-lative abundance.

To translate the metabolic differences noted using the data visualization methods, we chemically profiled the dysregulated metabolites by AgNPs aided by the CANOPUS software, a computational tool for systematic chemical classifications based on Classyfire’s chemical ontologies [29] using fragmentation spectra [30]. Figure 4 shows the dysregulated chemical classes assigned by CANOPUS, wherein carboxylic acids and fatty acyls represent the most altered chemistry in both lifeforms of *Vibrio cholerae* exposed to the nanoparticles. Other chemical classes were uniquely changed in each lifeform: organooxygen compounds, saccharolipids, and organonitrogen compounds were perturbed in the planktonic cells, whereas glycerophospholipids and azoles were increased in abundance in the biofilms.

We further identified or annotated at the molecular structure level the dysregulated metabolites by treatment using GNPS (Global Natural Products Social Molecular Networking web platform) automatic spectral matching (MSI, level 2) and *in silico* annotation tools (i.e., SIRIUS and MolDiscovery software, MSI, level 3). The complete list of all the putatively identified metabolites is shown in Tables S1–S3. 

**Figure 5:**
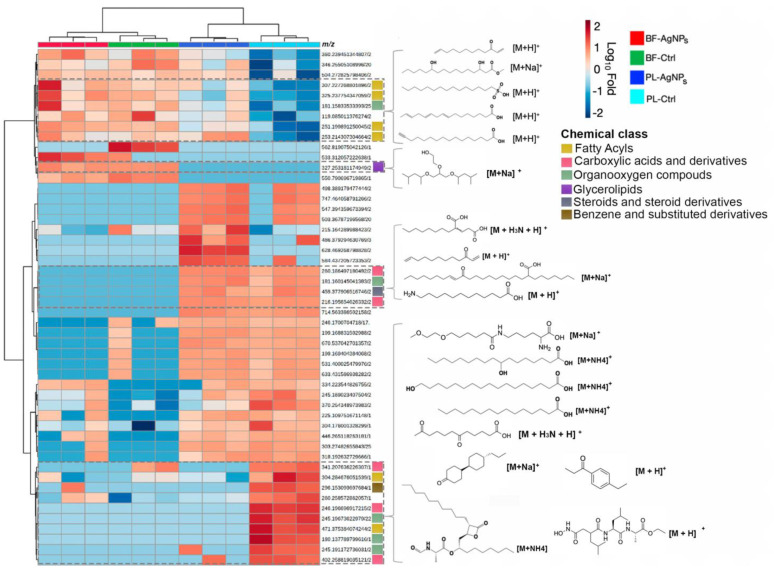
Heatmap and hierarchical clustering visualizations of the top 50 metabolites ranked by ANOVA, exhibiting the different metabolite abundances among the AgNP treatment and control groups. Chemical classes are shown in different colors, and on the right of the heatmap, the values of m/z of each metabolite are shown. Data were log-transformed without scaling for the heatmap analyses. The clustering method used was Ward, using Euclidean distance as a distance measure. (BF-AgNPs—Biofilm exposed to AgNPs; BF-Ctrl—Unexposed biofilm control; PL-AgNPs—Planktonic cells exposed to AgNPs; PL-Ctrl—Unexposed planktonic cells control; QC—Quality control.

We observed a subset of saturated and unsaturated fatty acids with relatively lower abundance in the planktonic cells compared to the biofilms, whose levels were only mo-dulated (i.e., increased) by AgNPs in planktonic cells (Figure 5). Likewise, but in the opposite direction, various glycolipids (glycosylated fatty acids), fatty acids (e.g., hydro-xylated and aminated), and amino acid derivatives with higher abundance in planktonic cells compared to biofilms were downmodulated by AgNPs in planktonic cells (Figure 5). The metabolic differences between the lifeforms were also corroborated (Figure 5), showing a characteristic abundance pattern for biofilms (treated and control) and the opposite for planktonic cells (treated and control). In Figure 5, the molecular structures denote the putative metabolite predicted by the CSI: FingerID tool in the SIRIUS software. The che-mical classes are shown in different colors, and on the right of the heatmap, the values of *m/z* of each metabolite are shown. Not all metabolites had an associated fragmentation spectrum for the *in silico* annotations, therefore it could not retrieve a chemical structure. 

**Figure 6:**
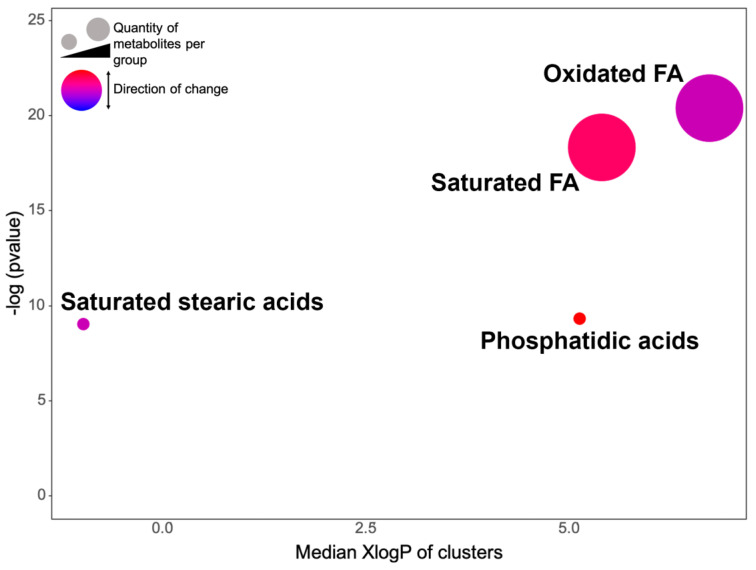
**Chemical enrichment analysis of the dysregulated metabolites in planktonic cells by silver nanoparticles (AgNPs).** Each node represents a significantly perturbed cluster of metabolites. Node sizes reflect the total number of compounds belonging to the chemical class. The node color scale shows the proportion of increased (red) or decreased (blue) compounds for each cluster. Different purple tones reflect the proportion of increased and decreased metabolites. *p*-values were calculated by the Kolmogorov–Smirnov test.

An enrichment analysis based on the chemical similarities was conducted to provide a comprehensive overview of the metabolites modulated by AgNPs. We only found enriched clusters of metabolites using the dysregulated metabolites by AgNPs in the planktonic cells including oxidized fatty acids (FA), saturated FA, phosphatidic, and saturated stearic acids (Figure 6).

## 3. Discussion

The evolution of multi-resistant microorganisms has promoted the search for new and different antimicrobial agents, as is the case with silver nanoparticles (AgNPs). Bacteria can exist as free-living organisms (i.e., planktonic cells) or form multilayer communities (i.e., biofilms), where the latter are associated with higher rates of drug resistance [8,31]. Although some studies report on the molecular effects of AgNPs on bacteria, few have provided molecular insight into the phenotype-dependent metabolic response to this nanomaterial. This is of the utmost importance for discovering new mechanisms leading to planktonic and biofilm eradication and understanding bacteria biology. In this study, we compared the antimicrobial effect of AgNPs in planktonic cells and biofilms of *V. cholerae* and gained insight into the bacterial phenotype-dependent metabolic response to the nanomaterial using mass spectrometry-based untargeted metabolomics and comprehensive chemoinformatics.

AgNPs were synthesized successfully with an average size of 10 to 35 nm, according to a size range of ≤100 nm, and a spherical shape was reported for silver nanoantibiotics [19,32,33]. The antimicrobial effect of AgNPs showed an evident difference in the range of the minimum bactericidal concentrations (MBCs) of the planktonic and biofilm lifeforms. Although the MBC for the planktonic cells of AgNPs was 550 µg/mL, the MBBC for the biofilms was 8.8 mg/mL. Regarding conventional antibiotics, multiple studies have shown a greater antimicrobial resistance of biofilms than planktonic cells [6,8,16,31,34,35,36]. Thus, a higher concentration of the same antibiotic is needed in the former to reach the desired activity [6,8,9,31,36,37]. Multiple resistance mechanisms have been described *in vitro*. For example, planktonic cells can regulate flagellar expression, promoting its survival in hostile environments and pathogenicity during cholera disease by expressing virulence genes [38], whereas biofilm communities have drug-modifying enzymes and drug-neutralizing proteins, lower cell permeability, efflux pumps, and low metabolism [6,14,19,39,40,41,42,43,44]. However, evidence also shows that AgNPs exert a multimodal antimicrobial activity. These mechanisms include (i) cell wall and membrane alterations, causing morphological changes such as changes in permeability and loss of stability; (ii) membrane chemical changes, especially in fatty acids, proteins, and carbohydrate compositions; (iii) interference in the respiratory chain due to their interaction with membrane proteins; and (iv) interruption of transcription, translation, and protein synthesis, leading to necrosis and death [14,18,19]. Nevertheless, the exposure of microorganisms to antimicrobials at sublethal concentrations or for short periods allows us to evaluate the changes in the exposed cells at the genetic, protein, and metabolic expression levels to assess their survival and probably selection of resistant traits. In that sense, we were interested in describing the metabolic responses of planktonic and biofilm cells to overcome AgNPs’ toxicity.

Based on the antimicrobial activity of AgNPs over *V. cholerae* planktonic and biofilm cells, a concentration equivalent to half the MIC was selected as the sublethal dose (275 µg/mL for planktonic cells and 2.2 mg/mL for biofilms). After 24 h of exposure to the sublethal dose, there was a high amount of viable biomass (Figure 2a,b) and the cells by the MTT assay maintained metabolic activity (cellular respiration) (Figure 2e,f). This allowed us to obtain and evaluate the expression of the metabolites from *V. cholerae* cells that were and were not exposed to AgNPs. A mass spectrometry-based untargeted metabolomic analysis approach was applied to assess the metabolic changes triggered by a sublethal concentration of AgNPs in the two lifeforms. To provide a global perspective of the meta-bolomes under each condition, we performed a PCA analysis [45]. Both the planktonic and biofilm control groups diverged, indicating differences in their basal metabolome (Figure 3a and Figure 5). These results agree with the published literature, where significant differences in community compositions, structures, and the metabolic activity of planktonic cells and biofilms have been described [21,46,47]. Our results showed that (i) biofilm exposed to AgNPs and the biofilm control group clustered together, showing a clear distinction from the planktonic groups (Figure 3a); (ii) more significant dysregulated meta-botalites were found in planktonic cells treated with AgNPs than in biofilm AgNPs-treated cells (Figure 3b–d); and (iii) only 3 of the 105 perturbed features were shared among the metabolomes from the planktonic cells and biofilms (Figure 3c). These results support the notion that AgNPs impact the microorganism’s metabolome depending on the microbial phenotype (Figure 3a and Figure 5).

Biofilm cells exposed to AgNPs and the biofilm control clustered together in the PCA (Figure 3a) and had a few dysregulated metabolites (10 down—23 up) (Figure 3b). We can attribute this to the previously described resistance phenomena of biofilms to antimicrobial agents or harsh environmental conditions, where the biofilms’ low growth rate (dormant cells) and metabolic heterogeneity can promote tolerance [31]. It has been reported that in biofilms due to the negatively charged exopolysaccharides (EPS) matrix composition, the slow diffusion of negatively charged compounds prevents their internalization [31]. Additionally, due to the surface charge of AgNPs, negatively charged and neutral AgNPs can be trapped by EPS or adhere to cell membranes, internalizing in small amounts, whereas positively charged AgNPs exhibit more efficient cell membrane internalization [48]. We can propose that cells within the biofilm interact with the AgNPs, supported by the observed changes in the metabolite abundance in the treated biofilms and their control on the heat map (Figure 5).

On the other hand, AgNPs-exposed planktonic cells differed from their control counterpart in the PCA diagram (Figure 3a), exhibited more dysregulated metabolites (35 up–36 down) (Figure 3d), and showed a specific abundance pattern for some metabolites (Figure 4). These results may be associated with their higher metabolic rate, growth speed, and direct contact with AgNPs in contrast to the biofilms [18,31,49]. It has been widely reported that planktonic bacteria are susceptible to the toxic effect of AgNPs. The main toxic mechanisms reported are (i) the cell wall and membrane alterations (permeability, gaps, and leakage of cellular contents), (ii) the intracellular oxidizing compounds, and (iii) the interaction with subcellular structures and biomolecules such as proteins and nucleic acids [18,31,49,50,51]. In this sense, the planktonic cells should respond with a more robust cellular mechanism to survive the toxic effects of AgNPs. 

Using GNPS automatic spectral matching we were not able to identify or annotate the metabolites dysregulated by AgNPs because the ones that were annotated were not found to be dysregulated. We, therefore, utilized a data analysis pipeline [52] that consisted of various advanced *in silico* annotation tools that allowed us to putatively annotate the metabolites at the molecular structure [53,54] and chemical class levels [30]. Various compound chemical classes were perturbed, and the abundance and diversity of the compounds were more significantly annotated in the planktonic cells than in the biofilms (Figure 4, Figure 5 and Figure 6). Since the metabolome diversity is specific for each lifeform and presents a particular chemical class abundance, we can conclude that the phenotype-dependent metabolic responses of *V. cholerae* are due to the AgNPs’ mode of action. According to our previous report [19], these results support the idea that AgNPs cause changes in the integrity of the cell membrane in *E. coli* and *S. enterica* biovar *Typhimurium*. However, sublethal concentrations of AgNPs depolarize the cell membrane, promoting an increase in permeability and making it more susceptible to bacteria against antimicrobial agents. Therefore, this change in the chemical composition of the membrane detected by LC-MS2 may suggest a possible survival mechanism when facing a sublethal concentration of AgNPs. The enrichment of this chemical class of compounds related to the formation or integrity of the cell membrane in planktonic cells and not in biofilm cells can be explained by the protective matrix formation of the biofilms, which delays and decreases the access of AgNPs to biofilm-forming cells. In contrast, planktonic cells are directly exposed to the AgNPs and greater susceptibility to the toxic effect of AgNPs is attributed to this direct contact [8,35,55,56]. 

In conclusion, this study shows the metabolomic profiles of planktonic cells and biofilms of *Vibrio cholerae* exposed to sublethal concentrations of AgNPs, demonstrating that there are more metabolic disturbances in the planktonic lifeform than in the biofilm lifeform. In the planktonic cells, carboxylic acids and fatty acids were mainly disturbed where, after a chemical enrichment analysis, a statistically significant abundance of fatty acids (saturated and unsaturated), phosphatidic, and saturated stearic acid were found, suggesting a membrane re-composition when exposed to sublethal concentrations of AgNPs. In addition, non-targeted metabolomics confirms the differences in the compositions and abundances of the specific metabolites in each lifeform of *Vibrio cholerae*. This work contributes a metabolomics perspective for exploring possible resistance pathways to AgNPs by *Vibrio cholerae*.

## 4. Materials and Methods

### 4.1. AgNPs Synthesis and Characterization 

AgNPs were synthesized following the methodology described by Vazquez-Muñoz [27]. Briefly, 30 mL of a 15 mM AgNO_3_ stock solution was warmed at 70 ± 5 °C on a stirring plate. Next, 5 mL of 30 mM PVP was added to the AgNO_3_ solution (maintained under vigorous stirring). Then, 300 µL of 4 mM of NaBH_4_ was added dropwise to the colorless solution until it turned a brown color. The suspension was vigorously stirred for an additional 10 min at 70 ± 5 °C. The AgNP synthesis was transferred to a light-protected plastic tube, cooled down at room temperature, and then stored at 4 °C. Then, the synthesis was dialyzed in PBS for 4 h (2 KDa molecular weight cutoff—MWCO) at 4 °C with continuous stirring to eliminate the remaining AgNO_3_. Finally, the synthesis was lyophilized to preserve its stability and storage at room temperature and make it light-protected. The synthesis was characterized by UV–Vis spectrophotometry (Multiscan GO, Thermo Scientific) and Dynamic Light Scattering (DLS) analysis (Zetasizer NanoSeries, Malvern) to obtain the hydrodynamic size and Z-potential. The analyses were conducted at the end of the synthesis, after dialysis and lyophilization, and before each treatment to corroborate the stability of the AgNPs. The silver concentration of the synthesis was calculated by a global chemical analysis performed by inductively coupled plasma-atomic emission spectroscopy (ICP-AES) (Variant Liberty 110 Spectrometer). The shape and size of the AgNPs were visualized by transmission electron microscopy (TEM) (Hitachi H7500).

### 4.2. Vibrio cholerae Growth and AgNPs Exposure 

*Vibrio cholerae* O1 El Tor (strain A1552 smooth variant) was used in this study. *V. cholerae* were cultured in 5 mL of Lysogeny Broth (1% tryptone, 1% sodium chloride, 0.5% yeast extract, pH 7.5) overnight at 30 °C and 200 rpm and used as pre-inoculum for further experiments. All treatments were performed with an initial bacterial inoculum of 1 × 10^4^ cell-forming units (CFU). Experiments were conducted in triplicate with at least three biological replicates.

### 4.3. Vibrio cholerae Planktonic Cells Antimicrobial Test

*V. cholerae* was grown in LB and exposed to different concentrations of AgNPs (from 9 µg/mL to 17.6 mg/mL) in a 96-well flat-bottom plate with a final volume of 200 µL per well for 24 h at 30 °C. After treatment, a 5 µL sample of each well was taken with a sterilized 96-tip stamper, transferred to an LB agar Petri dish, and incubated overnight at 30 °C. The minimum bactericidal concentration (MBC) was determined as the concentration at which there was no bacterial growth. A representative photograph was selected as e-vidence. Complementarily, the Minimal Inhibitory Concentration (MIC) was determined by measuring the bacterial growth by spectrophotometry at 600 nm in a microplate reader spectrophotometer, and an MTT assay (reducing the tetrazolium dye MTT 3- (4,5-dimethylthiazol-2-yl) -2,5-diphenyltetrazolium bromide to its insoluble formazan) was performed to validate viability by metabolic activity. A sublethal concentration of AgNPs was selected for the metabolomic analysis.

### 4.4. V. cholerae Biofilm Antimicrobial Test

*V. cholerae* biofilm was pre-formed and then treated with AgNPs. Briefly, 200 µL of the initial inoculum was inoculated in a 96-well flat-bottom plate and incubated for 48 h at 30 °C in static conditions to promote biofilm formation and maturation. Post-incubation, the planktonic cells were discarded and the 96-well plate was rinsed carefully with 200 µL PBS, leaving only the mature biofilm attached to the wells. A wide range of AgNPs concentrations (137 µg/mL to 35.2 mg/mL) in LB was selected for the treatments and then set in each well at a final volume of 200 µL. Cultures were incubated for 24 h at 30 °C under static conditions to not disturb the biofilm. A representative sample of each well was taken with a sterilized 96-tip stamper, stamped on an LB agar Petri dish, and incubated overnight at 30 °C. The minimal biofilm bactericidal concentration (MBBC), which is the minimal concentration of antimicrobial agent required to kill 99% of a pre-formed mature biofilm, was determined as the concentration at which no bacterial growth occurs. A representative photograph was selected as evidence. Additionally, the Minimal Inhibitory Concentration (MIC) was determined by measuring the bacterial growth by spectrophotometry at 600 nm in a plate reader spectrophotometer with the corresponding controls, and an MTT assay (reducing the tetrazolium dye MTT 3-(4,5-dimethylthiazol-2-yl)-2,5-diphenyltetrazolium bromide to its insoluble formazan) was performed to validate viability by metabolic activity.

### 4.5. Untargeted Metabolomics

#### 4.5.1. Microbial Growth 

To extract *V. cholerae* cell metabolites (metabolome), 50 mL of LB broth was inoculated with 125 µL of overnight culture, incubated at 30 °C, and agitated at 200 rpm. Growth was monitored by absorbance at 600 nm. When cell cultures reached an OD 600 of 0.3, 15 µL was used as inoculum for 15 mL of LB broth and incubated at 30 °C and 200 rpm. When the cell culture reached an OD 600 of 0.6 (considering that the culture homo-genized to the exponential growth phase), a sublethal concentration of 275 µg/mL of AgNPs was added and incubated for 30 min at 30 °C and 200 rpm. Then, aliquots of the treated bacterial and control cultures were harvested and washed 3x with PBS and centrifuged at 10,000× *g* for 10 min at room temperature to discard the secreted metabolites. The bacterial pellet was resuspended in PBS. Each culture was adjusted to 1.0 absorbance (600 nm) in 1 mL of PBS final volume in a clean tube for metabolite extraction. Samples were centrifuged at 10,000× *g* for 10 min at room temperature, the supernatant was removed, and the pellet was dried using a SpeedVac system and stored at −80 °C for further processing. 

For the biofilm, 2 mL of *V. cholerae* were cultured in a 24-well plate for 48 h at 30 °C under static conditions to promote biofilm formation and maturation (Initial inoculum at 1 × 10^4^ CFU/mL). A sublethal concentration of 2.2 mg of AgNPs was added and cells were exposed for 30 min at 30 °C. After that, cells were washed once with LB to remove the excess of AgNPs and planktonic cells. The biofilm was removed by pipetting up/down and then resuspended in 2 mL of PBS. The cultures were harvested and washed 3× with PBS (to remove media and metabolites inside the matrix). After the final wash, cells were centrifuged at 10,000× *g* for 10 min at room temperature. The subsequent steps were the same as those for the planktonic cells. The concentrations employed for both microbial phenotypes corresponded to half of the MIC based on the antimicrobial assays. We selected such concentrations and a short period of incubation (30 min) to observe changes at the metabolome level before cell death was considerable (i.e., 24 h).

#### 4.5.2. Metabolites Extraction 

The methodology described by Flores-Núñez was followed without modifications [57]. The dried samples were extracted with 500 µL of a mixture of methanol:acetonitrile:ethyl acetate at a 1:1:1 *v*/*v* ratio by sonication for 30 min at room temperature. Next, samples were centrifuged at 14,000 rpm for 10 min at 4 °C. The supernatant was recovered, transferred to a 1.5 mL Eppendorf tube, and deposited into a SpeedVac system to evaporate the solvent at room temperature. The dehydrated extracts were resuspended in a solution of water and acetonitrile (80:20 *v:v*), centrifuged at 14,000 rpm for 10 min at 4 °C, and the particle-free supernatant was recovered for more analysis. Equal volumes of all particle-free supernatants were mixed in one tube to make quality control (QC) samples.

#### 4.5.3. LC-MS2 Data Acquisition

We followed the instrumentation and protocol previously described [57] without modifications. The metabolites (2 µL sample injection) were separated in an Agilent nanoLC 1260 Infinity system (Agilent Technologies, Inc., Santa Clara, CA, USA) and a column packed with ZORBAX 80 SB-C18 (75 µm × 43 mm, 5 µm C18 with a 40 nL enrichment or trap column). Water and acetonitrile with 0.1% formic acid were used as mobile phases A and B, respectively, at a flow rate of 300 nL/min. The gradient started at 5% B, was increased linearly to 20% B in 20 min, was maintained at 20% B for 5 min, was increased linearly to 100%, was maintained at 100% B for 5 min, and was returned to 5% B in 1 min and then maintained at 5% B for 9 min before the following sample (to ensure column re-equilibration). Two blank samples (3 µL of mobile phases A and B at a 95:5 ratio) were run between the experimental sample injections to minimize potential carry-over. The flow from the column was deposited into a 6530 Accurate-Mass Q-TOF mass spectrometer (Agilent Technologies, Inc., Santa Clara, CA, USA) via an HPLC-Chip Cube MS interface. Nanospray ionization under positive mode was employed. MS data were acquired using the following conditions: capillary voltage, 1850 V; gas temperature, 350 °C; drying gas flow, 5 L/min; skimmer voltage, 65 V; octapole RF, 750 V; fragmentor voltage, 175 V; spectra acquisition rate, 4 spectra/s over a mass range of 110 to 2000 *m/z*. The MS2 data conditions were isolation window, narrow (1.3 *m/z*); spectra acquisition rate, 3 spectra/s; max precursors per cycle, 5 over a mass range of 50–2000 *m/z*. The active exclusion option was enabled, set to 2 spectra, and released after 0.25 min. The ramped collision energy (CE) option was used with slope and offset values of 6 and 4, respectively. The instrument was externally calibrated before sample acquisition using an ESI-L low-mix concentration tuning mix-solution (Agilent Technologies, Inc., Santa Clara, CA, USA) to ensure a mass accuracy of <5 ppm for both the MS and MS2 data. Instrument performance was supervised during data acquisition by including QC samples for every 4–5 experimental samples and evaluating the blank sample signals. Samples were randomly selected for data acquisition.

#### 4.5.4. LC-MS2 Data Processing and Analysis

The methodology was followed with some modifications from Flores-Nuñez [57]. Our approach consisted of analyzing the LC-MS2 datasets using open-access software packages and online platforms and following three fundamental approaches: (i) features were extracted and aligned among the datasets using MZmine version 2.53 data normalization [58], (ii) univariate and multivariate statistical analyses and hierarchical clustering visualization were performed using NormalyzerDE [59] and Metaboanalyst 4.0 [60], and (iii) automatic metabolite annotation and identification at the structure level were performed using the Global Natural Products Social Molecular Networking web platform (Metabolomics Standards Initiative [MSI] classification level 2) [61] and *in silico* tools (MolDiscovery, CSI: FingerID) (MSI, level 3) [54,55]. Assignment of chemical classes to the modulated metabolites (FC > 1.5/1 or < 1/1.5, *p*-value < 0.05 Limma test) (MSI, level 3) was carried out using the CANOPUS tool [31] integrated within SIRIUS software version 4.9.12 [62]. The list of differentially abundant metabolites among the treated vs. non-treated microbial lifeforms was analyzed by ChemRICH [63], a chemical similarity enrichment analysis online software for metabolomics datasets. The pipelines with the specific processing parameters can be found in the Supplementary Materials (Protocol S1); the descriptions of the software and web pages (Table S4) and statistical analysis (Table S5) are also available. 

#### 4.5.5. Data Availability

The raw datasets are available in the GNPS/MassIVE public repository with the accession number MSV000088670. The parameters for the classical molecular networking and spectral matching using all datasets are accessible at the following link: https://gnps.ucsd.edu/ProteoSAFe/status.jsp?task=db1a30deb7914986b876d09644eef080. (Accessed on 11 January 2022). The MolDiscovery results are available at the following link: https://gnps.ucsd.edu/ProteoSAFe/status.jsp?task=53eabc6b4fa34830925f2c0296ca0f4d. (Accessed on 03 March 2022).

#### 4.5.6. Metabolomics Statistical Analysis

We followed the methodology previously described [58] with some modifications. For the metabolomics data, features with reproducible quantification (based on QC coefficient of variation <25%) and a fold change ≥1.5 or ≤1/1.5 and a *p*-value < 0.05 (Limma test) were considered differentially abundant when comparing the treated vs. non-treated microbial lifeforms. Metaboanalyst 4.0 was utilized for the multivariate statistical analysis and heatmap visualization. Principal component analysis (PCA) was used to assess sample clustering behavior and inter-group variation. Log-transformed data (without scaling) were used for PCA and heatmap analysis.

## Data Availability

The raw datasets are available in the GNPS/MassIVE public repository with the accession number MSV000088670. The parameters for classical molecular networking and spectral matching using all datasets are accessible at the following link: https://gnps.ucsd.edu/ProteoSAFe/status.jsp?task=db1a30deb7914986b876d09644eef080. (Accessed on 11 January 2022). The MolDiscovery results are available at the following link: https://gnps.ucsd.edu/ProteoSAFe/status.jsp?task=53eabc6b4fa34830925f2c0296ca0f4d. (Accessed on 03 March 2022).

## Supplementary Materials

The following are available online at https://www.mdpi.com/article/10.3390/antibiotics11111534/s1, Figure S1: Dynamic light scattering analysis of AgNPs; Figure S2: Percentage weight calculation of AgNPs; Figure S3: MTT assay; Protocol S1: Detailed processing parameters for all the pipelines. Table S1: List of all the putatively annotated metabolites by MS2 spectral matching against GNPS public spectral libraries; Table S2: List of all the top putatively annotated metabolites by in silico tool SIRIUS 4.9.12 and the top chemical class; Table S3: List of all the putatively annotated metabolites by in silico tool MolDiscovery (1.0.0); Table S4: Description of software and web pages; Table S5: Description of statistical analysis. References [29,30,53,54,58,59,61,62,63,64,65,66,67,68,69,70] are cited in the supplementary materials.
