# Supplementary material for: Metabolomic Profiling of the Responses of Planktonic and Biofilm Vibrio cholerae to Silver Nanoparticles
Source: Antibiotics (Basel). 2022 Nov 2;11(11):1534. doi: 10.3390/antibiotics11111534 (PMC9686607; doi:10.3390/antibiotics11111534)
Supplement: Supplementary file 1 [file antibiotics-11-01534-s001.zip › Supplementary_antibiotics-1991930.pdf]

Supplementary Information

Metabolomic profiling of the response of planktonic and biofilm *Vibrio cholerae t*o silver nanoparticles

Anaid Meza-Villezcas[1-3¶], Rommel A. Carballo-Castañeda[2], Aldo Moreno-Ulloa[2], Alejandro Huerta-Saquero[1*]

[1] Departamento de Bionanotecnología, Centro de Nanociencias y Nanotecnología, Universidad Nacional Autónoma de México, Ensenada, Baja California, México.
[2] Departamento de Innovación Biomédica, Centro de Investigación Científica y de Educación Superior de Ensenada (CICESE), Baja California, México
[3] Departamento de Microbiología, Centro de Investigación Científica y de Educación Superior de Ensenada (CICESE), Baja California, México

* Corresponding author
E-mail: saquero@ens.cnyn.unam.mx

Supplementary Figures

S1 Figure. Dynamic Light Scattering analysis of AgNPs.

S2 Figure. Calculations of the AgNP concentration of the synthesis.

S3 Figure. Experimental visualization of the MTT assays.

Supplementary Tables

S1 Table. List of all the putatively annotated metabolites by MS2 spectral matching against GNPS public spectral libraries. (Available as excel file).

S2 Table. List of all the top putatively annotated metabolites by in silico tool SIRIUS 4.9.12 and top chemical class. (Available as excel file).

S3 Table. List of all the putatively annotated metabolites by in silico tool MolDiscovery (1.0.0). (Available as excel file).

S4 Table. Description of software and web-pages.

S5 Table. Description of statistical analysis.

Supplementary Protocol

S1 Protocol. Detailed processing parameters for all the pipelines.

S1 Figure. Dynamic Light Scattering analysis of AgNPs. (a) Hydrodynamic size distribution (nm) of the AgNP. (b) Zeta-potential to measure the charge repulsion/attraction between AgNP to know its stability.

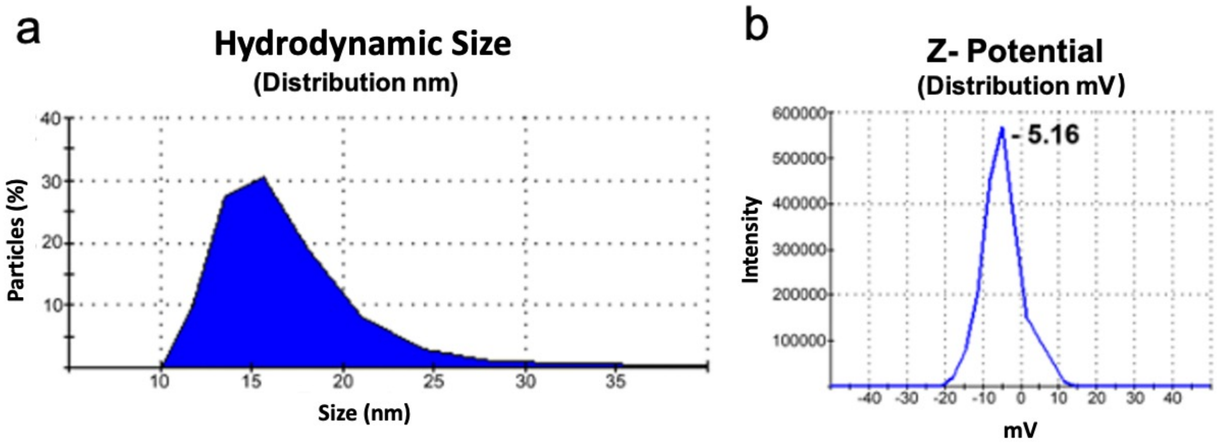

S2 Figure. Calculations of the AgNP concentration of the synthesis. (a) Formula to calculate the percentage weight. (b) Estimation of the AgNP percentage weigh in the synthesis.

a

$$\text{Wt \%} = \left[\frac{\textit{[Ag concentration by ICP-OES (ppm)]} \times \textit{[Dilution factor]}}{\textit{Weight of digested synthesis (mg)}} \right] \text{X } 100$$

b

$$\text{Wt \%} = \left[\frac{\textit{[1.8587 ppm]} \times \left[\frac{1}{50} \times \frac{1}{25} \right]}{\textit{2.6 mg}} \right] \text{X } 100 = 88\ \%$$

S3 Figure. Experimental visualization of the MTT assays. (a) Planktonic cells and (b) biofilm treated with wide range of AgNP concentrations.

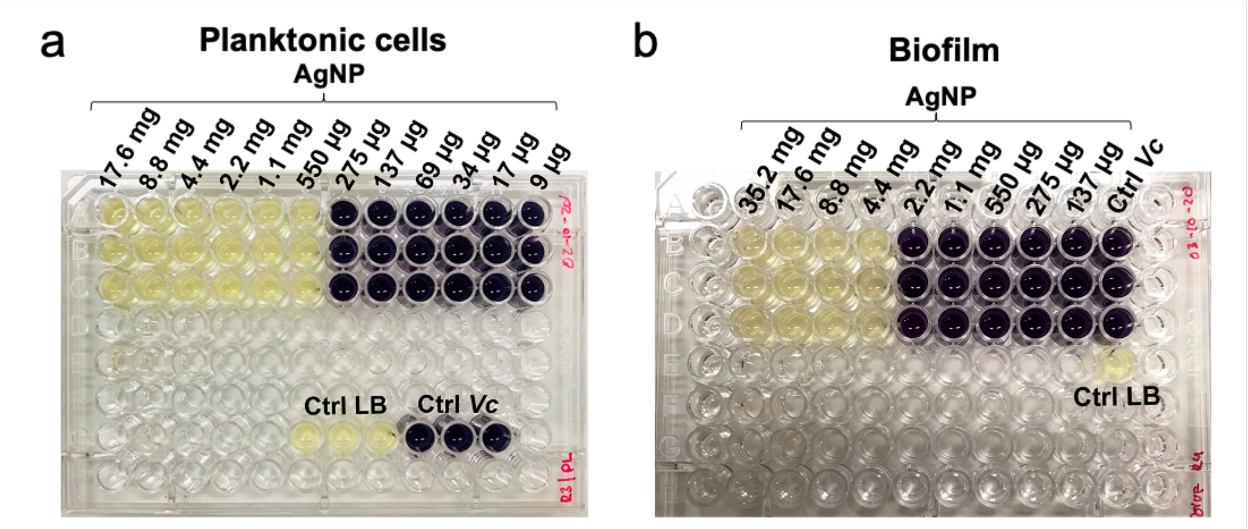

S4 Table. Description of software and web-pages used in this work.

Software	*Description*	*Ref **
**MZmine version 2.53**	An open-source software to process MS raw data for peak picking, assigning quantitative information to metabolites using features AUC, based on extracted chromatogram information.	[58]
**NormalyzerDE**	Online tool for selection of normalization method based on R programming language for statistical analysis of multivariable data.	[59]
**MetaboAnalyst 4.0**	Platform based on R programming language for multivariate and univariate statistical analysis of quantitative data.	[60]
**Global Natural Products Social Molecular Networking web platform (GNPS)**	Mass spectrometry-based metabolomics database constantly enriched and curated by users, adding metadata for reanalysis, and annotate compounds at different confidence levels.	[61]
**MolDiscovery**	Annotation tool which uses a graph-based *in-silico* fragmentation algorithm to assign molecular substructures in MS2.	[53]
**CSI: FingerID**	Annotation tool based on the construction and alignment of molecular fingerprints by predicting substructures in fragmentation trees.	[54]
**CANOPUS**	Machine learning-trained algorithm to assign a chemical classification to an MS2 based on molecular fingerprints constructed with CSI:FingerID.	[31]
**SIRIUS**	Reranking algorithm based on Gibbs sampling and Bayesian statistics for molecular formula annotation.	[62]
**ChemRICH**	Online tool for chemical enrichment analysis, a statistical test which shows chemical classes represented in a set of differentially abundant metabolites.	[63]

* References in the main text.

S5 Table. Description of statistical analysis.

Statistical Analysis	*Description*
**QC relative standard deviation > 25% filter**	Filtering step based on the variation of features abundance in the QC group. If the relative standard deviation is greater than a cutoff value (ex. 25% of the mean) the feature is removed from the matrix.
**Fold change ≥ 1.5 or ≤ 1/1.5**	Proportion of a features abundance relative to a control group.
**p-value < 0.05 Limma test**	The probability of obtaining the same measured value based on the premise of a correct null hypothesis.

S1 Protocol. Detailed processing parameters for all the pipelines.

For **approach #1**, proprietary raw.d files were converted to open-source .mzXML format with MSConvert version 3.0 [1] and then imported to MZmine 2.53 [2] for peak picking. Mass detection, chromatogram building and deconvolution (ADAP algorithm), isotope grouping, feature alignment, and gap filling (to detect features or peaks missed during the initial alignment) were performed to generate a list of potential metabolites or features. We discarded features from the list if: a) they were detected in the mobile phase and; b) they were present in less than two samples. The final filtered feature list or quantification table (peak areas aligned among groups) was exported as a .csv file for further statistical analysis and fragmentation patterns (MS2) in .mgf format for chemical class and compound annotation.

For **approach #2**, the quantification table was submitted to NormalyzerDE 1.5.4 [3] for normalization purposes (i.e., remove platform-specific sources of variability across samples). We chose the CycLoess normalization algorithm because it reduced the most inter-group and intra-group variation compared to the other algorithms available. Then, we exported the normalized feature's abundances list to Metaboanalyst 5.0 [4] to perform multivariate statistical analyses (PCA, Heatmap visualization). We filtered peaks with relative standard deviation (RSD) >25% in QC samples to retain high reproducible data. For PCA and Heatmap visualization, data was Log 2 transformed. Univariate statistical analysis was done using NormalyzerDE 1.5.4 [3] to determine the metabolites significantly perturbed by AgNPs treatment, using as cut-off values; a $p < 0.05$ (Limma package t test) and log2 fold change of ± 0.58. The overlapping pattern of the dysregulated metabolites was determined by Up-Set plotting using the upset R package [5].

For **approach #3**, .mzXML files were uploaded to the GNPS platform to perform Classical Molecular Networking [6] and automated structural annotation (against GNPS public spectral libraries) (MSI classification, level 2) [7]. To expand the putative annotation of metabolites (MSI classification, level 3) not achieved by spectral matching, we employed the MolDiscovery [8] and SIRIUS [9] *in-silico* dereplication tools through the GNPS platform and SIRIUS GUI version 4.9.12, respectively. For MolDiscovery, the clustered .mgf file retrieved from Classical Molecular Networking was analyzed. Parameters were as follows: precursor and fragment ion mass tolerance, 0.02 Da; max charge, 2; predefined DB, AllDB (720K compounds). For SIRIUS, we used the .mgf file from MZmine. First, we determined the elemental composition of peaks using SIRIUS and reranked them with the ZODIAC algorithm [10]. Then, for *in-silico* structure annotation, we used CSI:FingerID [11]. Elemental composition and structural annotation were done using information from biological databases (Bio Database option) and establishing possible adducts; proton, sodium, potassium, ammonium, and water-loss. The chemical class assignment was done through CANOPUS [12], based on the chemical ontology from ClassyFire [13].

To speed up running times, we selected only compounds <850 Da. Common contaminants (e.g., plasticizer, silanes), boron-containing, and low mass accuracy >10 ppm annotations were removed from the analysis. Molecular structures were drawn using ChemDraw Professional version 16.0.1.4. We performed a chemical enrichment analysis by ChemRICH [14] using the annotated perturbed metabolites to translate the altered metabolome to biochemical processes. This analysis uses the MeSH ontology [15] for enriched chemical class annotation. To observe if the AgNPs induced the same biochemical changes on both *Vibrio* lifeforms, we compared the chemical class profiles of the perturbed features by treatment in both lifeforms, using UpSet plotting with the complexUpset package in R [16].

References

1. Holman, J.D.; Tabb, D.L.; Mallick, P. Employing ProteoWizard to Convert Raw Mass Spectrometry Data. *Curr Protoc Bioinformatics* **2014**, doi:10.1002/0471250953.bi1324s46.
2. Olivon, F.; Grelier, G.; Roussi, F.; Litaudon, M.; Touboul, D. MZmine 2 Data-Preprocessing to Enhance Molecular Networking Reliability. *Anal Chem* **2017**, *89*, 7836–7840, doi:10.1021/acs.analchem.7b01563.
3. Willforss, J.; Chawade, A.; Levander, F. NormalyzerDE: Online Tool for Improved Normalization of Omics Expression Data and High-Sensitivity Differential Expression Analysis. *J Proteome Res* **2019**, *18*, 732–740, doi:10.1021/acs.jproteome.8b00523.
4. Pang, Z.; Chong, J.; Zhou, G.; de Lima Morais, D.A.; Chang, L.; Barrette, M.; Gauthier, C.; Jacques, P.É.; Li, S.; Xia, J. MetaboAnalyst 5.0: Narrowing the Gap between Raw Spectra and Functional Insights. *Nucleic Acids Res* **2021**, *49*, W388–W396, doi:10.1093/nar/gkab382.
5. Conway, J.R.; Lex, A.; Gehlenborg, N. UpSetR: An R Package for the Visualization of Intersecting Sets and Their Properties. *Bioinformatics* **2017**, *33*, 2938–2940, doi:10.1093/bioinformatics/btx364.
6. Aron, A.T.; Gentry, E.C.; McPhail, K.L.; Nothias, L.F.; Nothias-Esposito, M.; Bouslimani, A.; Petras, D.; Gauglitz, J.M.; Sikora, N.; Vargas, F.; et al. Reproducible Molecular Networking of Untargeted Mass Spectrometry Data Using GNPS. *Nat Protoc* **2020**, *15*, 1954–1991, doi:10.1038/s41596-020-0317-5.
7. Schymanski, E.L.; Jeon, J.; Gulde, R.; Fenner, K.; Ruff, M.; Singer, H.P.; Hollender, J. Identifying Small Molecules via High Resolution Mass Spectrometry: Communicating Confidence. *Environ Sci Technol* 2014, *48*, 2097–2098.
8. Cao, L.; Guler, M.; Tagirdzhanov, A.; Lee, Y.Y.; Gurevich, A.; Mohimani, H. MolDiscovery: Learning Mass Spectrometry Fragmentation of Small Molecules. *Nat Commun* **2021**, *12*, doi:10.1038/s41467-021-23986-0.
9. Dührkop, K.; Fleischauer, M.; Ludwig, M.; Aksenov, A.A.; Melnik, A. v.; Meusel, M.; Dorrestein, P.C.; Rousu, J.; Böcker, S. SIRIUS 4: A Rapid Tool for Turning Tandem Mass Spectra into Metabolite Structure Information. *Nat Methods* **2019**, *16*, 299–302, doi:10.1038/s41592-019-0344-8.
10. Ludwig, M.; Nothias, L.F.; Dührkop, K.; Koester, I.; Fleischauer, M.; Hoffmann, M.A.; Petras, D.; Vargas, F.; Morsy, M.; Aluwihare, L.; et al. Database-Independent Molecular Formula Annotation Using Gibbs Sampling through ZODIAC. *Nat Mach Intell* **2020**, *2*, 629–641, doi:10.1038/s42256-020-00234-6.
11. Dührkop, K.; Shen, H.; Meusel, M.; Rousu, J.; Böcker, S. Searching Molecular Structure Databases with Tandem Mass Spectra Using CSI:FingerID. *Proc Natl Acad Sci U S A* **2015**, *112*, 12580–12585, doi:10.1073/pnas.1509788112.
12. Dührkop, K.; Nothias, L.F.; Fleischauer, M.; Reher, R.; Ludwig, M.; Hoffmann, M.A.; Petras, D.; Gerwick, W.H.; Rousu, J.; Dorrestein, P.C.; et al. Systematic Classification of Unknown Metabolites Using High-Resolution Fragmentation Mass Spectra. *Nat Biotechnol* **2021**, *39*, 462–471, doi:10.1038/s41587-020-0740-8.
13. Djoumbou Feunang, Y.; Eisner, R.; Knox, C.; Chepelev, L.; Hastings, J.; Owen, G.; Fahy, E.; Steinbeck, C.; Subramanian, S.; Bolton, E.; et al. ClassyFire: Automated Chemical Classification with a Comprehensive, Computable Taxonomy. *J Cheminform* **2016**, *8*, 1–20, doi:10.1186/s13321-016-0174-y.

14. Barupal, D.K.; Fiehn, O. Chemical Similarity Enrichment Analysis (ChemRICH) as Alternative to Biochemical Pathway Mapping for Metabolomic Datasets. *Sci Rep* **2017**, *7*, doi:10.1038/s41598-017-15231-w.
15. Lowe, H.J.; Octo Barnett, G. *Understanding and Using the Medical Subject Headings (MeSH) Vocabulary to Perform Literature Searches*;
16. Michał Krassowski, M.A.& C.Lagger. Krassowski/Complex-Upset: V1.3.3 (v1.3.3) 2021.
